# Intentional interruptions during compression only CPR: A scoping review

**DOI:** 10.1016/j.resplu.2024.100623

**Published:** 2024-04-04

**Authors:** Giulia Catalisano, Marta Milazzo, Barbara Simone, Salvatore Campanella, Francesca Romana Catalanotto, Mariachiara Ippolito, Antonino Giarratano, Enrico Baldi, Andrea Cortegiani

**Affiliations:** a Department of Precision Medicine in Medical Surgical and Critical Care (Me.Pre.C.C.), University of Palermo, Italy; b Department of Anesthesia Intensive Care and Emergency. University Hospital Policlinico ‘Paolo Giaccone’, Palermo, Italy; c Division of Cardiology, Fondazione IRCCS Policlinico San Matteo, Pavia, Italy; d Cardiac Arrest and Resuscitation Science Research Team (RESTART), Fondazione IRCCS Policlinico San Matteo, Pavia, Italy

**Keywords:** Out of Hospital Cardiac Arrest (OHCA), CPR interruptions, Pauses during CPR, Continuous Chest Compressions (CCC), Compression only CPR (CO-CPR)

## Abstract

**Introduction:**

Out of hospital cardiac arrest (OHCA) remains one of the main causes of death among industrialized countries. The initiation of cardiopulmonary resuscitation (CPR) by laypeople before the arrival of emergency medical services improves survival. Mouth-to-mouth ventilation may constitute a hindering factor to start bystander CPR, while during continuous chest compressions (CCC) CPR quality decreases rapidly. The aim of this scoping review is to examine the existing literature on strategies that investigate the inclusion of intentional pauses during compression-only resuscitation (CO-CPR) to improve the performance in the context of single lay rescuer OHCA.

**Methods:**

The protocol of this Scoping review was prospectively registered in Open Science Framework (https://osf.io/rvn8j). A systematic search of PubMed, Scopus, EMBASE, CINAHL was performed.

**Results:**

Six articles were included. All studies were carried out on simulation manikins and involved a total of 1214 subjects. One study had a multicenter design. Three studies were randomized controlled simulation trials, the rest were prospective randomized crossover studies. The tested protocols were heterogeneous and compared CCC to CO-CPR with intentional interruptions of various length. The most common primary outcome was compressions depth. Compression rate, rescuers’ perceived exertion and composite outcomes were also evaluated. Compressions depth and perceived exertion improved in most study groups while compression rate and chest compression fraction remained within guidelines indications.

**Conclusions:**

In simulation studies, the inclusion of intentional interruptions during CO-CPR within the specific scenario of single rescuer bystander CPR during OHCA may improve the rate of compressions with correct depth and lower rate of perceived exertion. Further high-quality research and feasibility and safety of protocols incorporating intentional interruptions during CO-CPR may be justified.

## Introduction

The annual incidence of Out of Hospital Cardiac Arrest (OHCA) is estimated between 30.0 and 97.1 individuals per 100,000 population with consistent values among years and countries.^1^ OHCA is burdened by high mortality, remaining one of the main causes of death among industrialized countries.^1^, ^2^

The vast majority of OHCA happens at home or in residential contexts and it is reported that individuals receiving cardiopulmonary resuscitation (CPR) by witnesses while waiting for emergency medical services (EMS) are almost twice as likely to survive the event.^3^, ^4^, ^5^, ^6^ Even so, the percentage of people receiving bystander CPR is low with great geographical variability ranging from 13% to 82% in Europe and 40% in the United States (US).^1^, ^3^, ^4^, ^7^ Since CPR performed by bystanders has a proven impact on outcomes, several studies aimed to understand the underlying factors that may hinder the immediate starting of CPR. Socioeconomic, racial and gender variables have been examined, resulting in a decreased likelihood of Black and Hispanic people of receiving bystander CPR in the US. An enhanced percentage of CPR started in male patients compared to female patients was also observed. 4, 8, 9, 10 The bystander’s fear of infection or personal injury is greatly linked to the presence of mouth-to-mouth ventilations in the CPR protocol, increasing their reluctancy to start CPR when the victim is a stranger. Moreover, this fear may have been exacerbated during the recent COVID-19 pandemic.^4^, ^11^, ^12^, ^13^, ^14^, ^15^, ^16^, ^17^, ^18^

As an alternative to standard CPR the International Liaison Committee on Resuscitation (ILCOR) and other societies have introduced the possibility of performing continuous chest compressions withholding mouth-to-mouth ventilations from the basic life support algorithm for adult patients if the rescuer does not feel safe to perform them or is not trained to do so, with the aim of favoring the initiation of chest compressions.^19^ Surveys and studies addressing the topic consistently report that in the case of unknown victims lay rescuers prefer performing compression-only resuscitation.^11^, ^15^ Performing continuous chest compressions may be physically demanding and may lead to a worsening in compressions depth, rate, and general efficacy.^20^, ^21^

The aim of this scoping review is to investigate the existing literature on alternative strategies to continuous chest compressions that examine the inclusion of intentional pauses during compression-only resuscitation to improve the performance in the context of single lay rescuer OHCA.

## Methods

### Protocol and search strategy

The protocol of this Scoping review was prospectively registered in Open Science Framework (https://osf.io/rvn8j). We performed a systematic search of PubMed, EMBASE, CINAHL and Scopus from inception until the 29th of January 2024 for randomized controlled trials, non-randomized trials, prospective and retrospective observational studies and abstracts addressing the introduction of intentional interruptions during compression-only CPR on patients, animals or training manikins. Studies investigating mandatory interruptions of CPR related to the use of automated external defibrillators were excluded. Case reports and conference proceedings were excluded as well. No language restriction was applied to the search. The search strategy included keywords as exact phrases and as combination of broad subject headings, according to database syntax provided in Supplementary Material S1.

### Inclusion and exclusion process

**Fig. 1:**
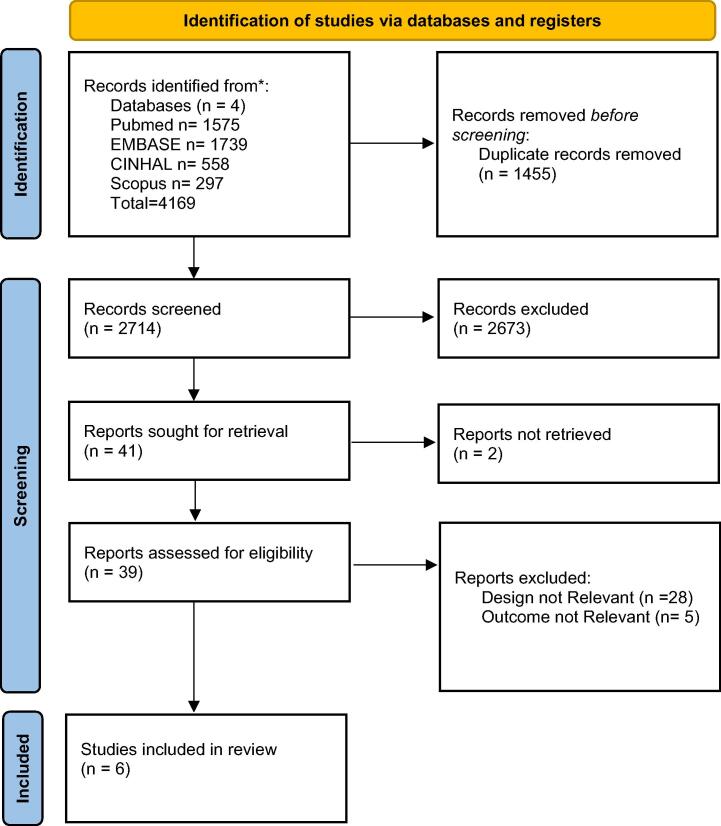
PRISMA 2020 flow diagram for new systematic reviews which included searches of databases and registers only.

All the retrieved records were divided into two halves. Each half was independently screened by two authors (SC and MM or BS and FRC) from title and abstract. The selected records were then independently reviewed from full text by the same two authors, to verify the fulfillment of the inclusion criteria. Studies were included if the screening authors agreed regarding eligibility. Disagreements at any stage were adjudicated by a fifth author (GC). Snowballing search on the references of selected articles was also performed. Data extraction was performed by one author (GC) and checked independently by two authors (MI, AC). This scoping review was structured following the Preferred Reporting Items for Systematic Reviews and Meta-Analysis: extension for Scoping Reviews (PRISMA-ScR) as presented in the flow diagram (Fig. 1) and checklist (Supplementary Material S2).^22^

## Results

### Characteristics and population of the studies

A total of 4169 records were retrieved in the comprehensive search. After the exclusion of duplicates and not relevant records, six studies were included in the scoping review. The process of inclusion and exclusion is detailed in the PRISMA-ScR diagram, provided in Fig. 1.^22^ Six full paper articles 23, 24, 25, 26, 27, 28 were retrieved. The characteristics of the included studies are provided in Table 1. One study had a multicenter design^23^. Half of the studies were designed as randomized controlled simulation trials^23^, ^26^, ^27^, while the other half had a prospective randomized crossover design^24^, ^25^, ^28^. No studies involving human patients or animals were retrieved. All studies were based on simulation manikins. A total of 1214 subjects were involved in the simulations. In three studies, participants were volunteer laypeople^23^, ^24^, ^26^, in one study participants were firefighters^25^, and in the remaining two studies participants were nursing students and emergency medical technician trainees^27^, ^28^. Three studies considered compression depth as the primary outcome^23^, ^24^, ^28^. Compression rate and composite outcome scores were also used to assess the interventions’ efficacy^26^, ^27^. The duration of the scenarios varied between 8 minutes^23^, ^26^ and 10 minutes^24^, ^25^, ^28^, with an exception in the study by Lim et al. where the scenario lasted 5 minutes^27^. For each study, several interventions were compared (Table 1).

**Table 1:** Characteristics of the studies included in the scoping review.

Authors (year)	Design (Country)	Setting	Population	Interventions	Outcomes	Results
Baldi et al (2020)	Multicenter International Randomized Control Study (Italy, Switzerland)	Scenario: OHCA Simulation of singlerescuer CPR on a training manikin (Laerdal Resusci Anne QCPR) .	Subjects: 576 volunteer laypeople Training: BLS/AED course according to ILCOR 2015 recommendations with 1-minute of CO-CPR without visual feedback reaching a result of ≥ 75% in the chosen parameters during the preliminary test. Allocation: randomization 1:1:1:1 in the four study groups	1)30c2s: 30 compressions 2 s rest for 8 min 2)50c5s: 50 compressions 5 s rest for 8 min 3)100c10s: 100 compressions 10 s rest for 8 min 4) CCC-CPR for 8 min	Primary outcome: Percentage of compressions with correct depth Secondary outcomes: Percentage of compressions -correctly released -with correct hand positioning -with adequate rate Interruptions for more > 10 s Chest compression fraction Assessment: Laerdal QCPR software	The groups performing 30c2s and 50c5s had a higher rate of compressions with correct depth. The CCF was higher in the CCC-CPR group There was no difference in the number of compressions correctly released and with adequate rate among the groups
Chang et al (2021)	Single center, prospective, randomized crossover study (Taiwan)	Scenario: OHCA Simulation of EMT long-term CPR on a training manikin (Laerdal Little Anne QCPR)	Subjects: 70 male firefighters Training: Emergency Medical Technician Intermediate licenses or higher and field experience Allocation: participants completed 3 CPR quality tests in random order with at least 2 days rest between each test	1)CCC CPR for 10 min 2)CPR-10 s: 5 sets of 2 min compressions separated by 10 s rest 3)CPR-20 s: 5 sets of 2 min of compressions separated by 20 s rest	Primary outcome: Correlation between body composition and CPR quality Secondary outcome: Comparison of CPR quality with different chest compressions interruption times Assessment: Laerdal SimPad PLUS device and SkillReporter extractor software	CCF and CCRR were lower in CCC CPR than in CPR-20 s method RPE values were higher in CCC CPR than in CPR-10 s and CPR-20 s methods Chest compression depth or rate did not differ among the three methods With the increase of total chest compression time CPR-20 s method saw a decrease in less CPR quality index parameters than CPR-10 s
Dong et al (2021)	Single center, Randomized crossover study (China)	Scenario: OHCA Simulation of single rescuer CPR on a training manikin (Resusci Anne, Laerdal Medical)	Subjects: 30 volunteer laypeople Training: “WeCan CPR” video-based 1 h course with 1-minute of CPR on a manikin without feedback reaching a score ≥ 75%. Allocation: participants were randomly divided in 5 groups and allocated to test the 5 methods in different order with at least 12 h rest between two tests.	1) CCC for 10 min 2) 4 + 6: 4 min CCC + 6 min of 60 compressions and 10 s rest 3) 2 + 8, 10/60: 2 min CCC + 8 min of 60 compressions and 10 s rest 4) 2 + 8, 5/30: 2 min CCC + 8 min of 30 compressions and 5 s rest 5)2 + 8, 3/15: 2 min CCC + 8 min of 15 compressions and 3 s rest	Primary outcome: Avarage chest compression depth Secondary outcomes: CPR quality indicators and participants’ fatigue indicators Assessment: Laerdal SimPad PLUS device and SkillReporter extractor software	CCF was higher in the CCC method 4 + 6 method showed shorter hands-off time and CCF than the 2 + 8 RPE was greater in the CCC method than in the 4 + 6 and 2 + 8. The quality of compression depth was the highest in 3/15 method, followed by the 10/60 and 5/30 method
Lim et al (2016)	Single center, Randomized control trial (Singapore)	Scenario: OHCA Simulation of single rescuer CPR on a training manikin (Laerdal Resusci Anne Skill reporter)	Subjects: 347 first year nursing students Training: participants were instructed with one of the two methods (CVC vs CCC) and tested again 6 months post-training Allocation: randomization in one of the two training programs and sub-randomization in 2 different landmark techniques for chest compressions.	1) CVC (Conventional CPR): 30 compressions 2 ventilations for 5 min 2) CCC: 100 compressions for 2 cycles followed by 10 s rest for 5 min	Primary outcome:rate of chest compressions (not including pause for ventilation and rest) and average number of compressions per minute (including pause for ventilation, rest) in two CPR training methods Assessment: Resusci-Anne manikin with skill meter.	The average compression rate was the same in CVC and CCC groups during the immediate testing and the 6-months post-training test. During the immediate testing and after 6 months, CVC groups did significantly fewer chest compressions per minute. All four groups had lower rates and did significantly fewer chest compressions per minute in the 6-months post-training test.
Min et al (2013)	Single center, Prospective, randomized crossover study (Republic of Korea)	Scenario: OHCA Simulation of single rescuer CPR on a training manikin (Laerdal Resusci Anne Skill reporter)	Subjects: 63 emergency medical technician trainees Training: a 120-min program consisting of CCC-CPR based on the 2010 guidelines Allocation: participants were divided into 3 groups. Each group was randomly assigned the three methods in different orders. Each method was performed more than 3 days apart.	1) CCC for 10 min 2) 200 compressions 10 s rest for 10 min (10/200) 3) 100 compressions 10 s rest for 10 min (10/100)	Primary outcome: Mean chest compression depth Secondary outcomes: Proportion of chest compressions with appropriate depth among total chest compressions during each minute Total number of compressions delivered per minute Compression rate Number and length of rest breaks Assessment: Resusci Anne manikin Skill Reporter	The 10/100 method showed the deepest compression depth, followed by the 10/200 and CCC methods; significant difference was observed after the 5th minute had elapsed. The 10/100 method showed the highest percentage of adequate compressions. The total number of chest compression over time was much higher during the CCC method.
Rasmussen et al (2016)	Single center, randomized controlled simulation study (Denmark)	Scenario: OHCA Simulation of single bystander rescuer DA-CPR on a training manikin (AMBU Man W)	Subjects: 128 volunteer laypeople Training: No previous CPR training in the past 2 years Allocation: randomization 1:1	1)Standard DA-CPR protocol including CCC-CPR for 8 min 2)Novel DA-CPR protocol including compressions and 10 s rest each minute for 8 min	Primary outcome: composite outcome score based on time to first compression, hand position, chest compression depth and rate and hands-off time Secondary outcomes: time to first compression, chest compression depth and rate, hand position and hands-off time Assessment: AMBU CPR Software	The novel protocol significantly improved composite outcome score compared with the standard protocol The novel protocol group had shorter time to first compression, better hand position and performed deeper and faster chest compressions compared with the control group: Compressions rate and depth were performed within guideline recommendations in both groups. Participants guided with the novel protocol had longer hands off time but maintained sufficient chest compression depth for all eight minutes.

Abbreviations: AED automated external defibrillator, CCC continuous chest compressions, CCF chest compression fraction, CCRR chest compression rebound rate, CO-CPR compression only-cardiopulmonary resuscitation, CPR cardiopulmonary resuscitation, DA dispatcher assisted, ILCOR International Liaison Committee on Resuscitation, OHCA out of hospital cardiac arrest, RPE rating of perceived exertion.

### Simulation protocols and outcomes

The biggest multicenter RCT was conducted by Baldi et al. including 576 volunteer laypeople and compared the inclusion of intentional pauses during compression-only CPR (CO-CPR) in an 8-minute scenario^23^. The percentage of compressions with correct depth was higher in the groups that performed 30 compressions followed by 2 s rest (96% (61.3–99.4)) and 50 compressions followed by 5 s rest (96% (63–100)) versus 100 compressions plus 10 s rest (92% (55–100)) and CO-CPR (79% (29.1–100%)) (p = 0.006). The single-center randomized controlled simulation study by Rasmussen et al. involved 128 volunteer laypeople and compared CO-CPR for 8 min with a novel protocol consisting of 1 min of chest compressions followed by 10 s of rest for 8 min. ^26^ In addition, modifications to the standard dispatch instructions given on speaker phone to bystanders starting cardiopulmonary resuscitation were applied.^26^ The primary outcome was a composite score including time to first compression, hands positioning, depth and rate of chest compressions and hands-off time, resulting in a better composite score for the intervention group with deeper and faster compressions, better hand position and shorter time to first compression, but with longer hands-off time. Participants guided with the novel protocol maintained sufficient chest compression depth for all eight minutes, while there was a decrease to less than 5 cm among participants guided with the standard protocol (p less than 0.05). The study by Dong et al. involving laypeople volunteers reported a higher quality of compression depth in the intervention consisting of 2 min of CO-CPR followed by 8 min of 15 chest compressions and 3 s rest, but longer hands-off time and lower chest compression fraction (CCF) for this technique.^24^ This study also evaluated the rating of perceived exertion which was greater for the continuous chest compression method. The perceived exertion was also evaluated by Chang et al. in a prospective randomized crossover study involving 70 firefighters in a 10-minute scenario, finding a higher rate of perceived exertion in the continuous chest compression technique compared to two other techniques consisting of 5 sets of 2 min chest compressions followed by 10- and 20-seconds rest respectively.^25^ The latter technique also allowed a lower decrease in CPR quality with the increase in total chest compression time and higher CCF values (97.08 ± 3.32%) compared to uninterrupted CPR (92.70 ± 9.49%). In a court of 63 emergency medical technician trainees, Min et al. found that performing 100 chest compressions followed by 10 s rest for 10 min compared to continuous chest compressions resulted in higher compression depth especially after the 5th minute had elapsed, with the highest percentage of adequate compressions.^28^ Lastly the study by Lim et al. was the only one comparing a technique with intentional interruptions (100 compressions for 2 cycles followed by 10 s of rest for 5 min) with conventional CPR (30 compressions, 2 ventilations) finding similar compression rate in the two groups but higher compression number in the intervention group.^27^

## Discussion

The feasibility and efficacy of introducing intentional interruptions during compression-only resuscitation by single bystander rescuer have been tested in simulation studies on manikins. The design of these studies is often single-centered and randomized but not controlled, implying a low level of evidence. The lack of studies in this area could be justified by the strong recommendation of guidelines against interruptions during CPR.^19^, ^29^ In fact, unintentional interruptions during chest compressions occur for a variety of reasons such as provider fatigue, switching of compressors, performance of ventilations, placement of invasive airways, application of CPR devices, pulse and rhythm checks, vascular access placement, and patient’s transfer to the ambulance.^29^ According to Sutton et al. up to 41.2% of the no flow time was related to switching compressors.^30^ The detrimental effect of the no-flow time is linked to the absence of a cardiac output produced by external chest compressions and consequently to the drop of systemic blood pressure, coronary pressure of perfusion and cerebral perfusion, resulting in lower rates of return of spontaneous circulation (ROSC).^29^, ^31^ It is therefore important to underline that in the simulation studies retrieved the scenarios are consistent, applying protocols in the specific event of OHCA where single rescuer bystander CPR is required while waiting for the EMS arrival. The rationale of introducing intentional interruptions could only be justified by the absence of another rescuer available to switch compressors, to maintain the highest possible CPR performance until the EMS arrival. In fact, shallow compressions are physiologically indistinguishable from the absence of chest compressions, mirroring the same outcomes with poor quality CPR.^32^ Therefore, it is of the utmost importance to identify the context in which the protocols examined in this scoping review may be applied.

The study protocols identified in the included studies vary in duration of the simulations, considered cohort (laypeople vs healthcare workers), number or minutes of compressions performed before the intentional interruptions and length of these. Even so, the results of the existing literature may suggest some advantages in the introduction of intentional pauses in the examined context.

The selected scenarios lasted either 8 or 10 min, except for Lim et al. study. This data is in line with the timing found in other studies. The report from the ILCOR on OHCA across the World recorded EMS response time as the interval from incoming call to the time the first emergency response vehicle stopped at the scene, with median intervals ranging from 6 to 13 min, and most registries reporting between 7 and 9 min.^1^, ^7^ During the COVID-19 pandemic time from OHCA to ambulance arrival was longer than in previous available data.^12^, ^33^ Therefore it is reasonable to protract the scenarios for this long. Moreover, the differences in CPR quality between CCC and other techniques were more marked the longer the scenarios lasted and according to Min et al. after the fifth minute elapsed. ^28^ This finding might be explained by the fact that perceived exertion was lower in protocols with interruptions, suggesting a lower grade of fatigue even when CRP is prolonged.^24^.

An important indicator of high-quality CPR is compression depth. Compression depth was observed to decrease with time during CO-CPR.^34^, ^35^ The introduction of intentional pauses has shown to improve the percentage of compressions performed with correct depth in four of the included studies, even if different protocols were used.^23^, ^24^, ^26^, ^28^ According to the findings of Baldi et al. the percentage of compressions performed with correct depth was greater in the groups that performed 30 compressions plus 2 s pause and 50 compressions plus 5 s pause.^23^ This finding is in line with the one by Dong et al. where the best compression depth was obtained when 2 min of continuous chest compressions were followed by 8 min in which the rescuer had to perform 15 compressions plus 3 s rest.^24^ The study showed improved rate of compression depth also for the groups that performed 60 compressions 10 s rest and 30 compressions 5 s rest. These findings are consistent with the one of Min et al. where the strategy of 100 compressions followed by 10 s rest provided deeper compressions.^28^ However, the same strategy did not translate into improved depth of compressions in the study by Baldi et al.^23^ These differences could be explained by the selection of the included subjects as Baldi et al. and Dong et al. included laypeople volunteers while Min et al. population was made of emergency medical technician trainees with experience in real cardiac arrest treatment.^23^, ^24^, ^28^

Another indicator of high-quality CPR is the compression rate which resulted within guidelines in all the protocols. CCF has been introduced as an adjunctive parameter to consider for high-quality CPR. CCF gives information about the proportion of time spent performing chest compressions during a resuscitation. The evidence is still debated as some studies highlight how higher CCF is associated with better outcomes while others show evidence of an inverse correlation between survival and CCF.^36^, ^37^, ^38^, ^39^ According to the American Heart Association latest guidelines on CPR high-performing EMS systems should target at least 60% of CCF, with 80% or higher being a frequent goal.^19^ CCF was evaluated in four of the included studies.^23^, ^24^, ^25^, ^26^ Data showed higher CCF in the CCC groups, except the study by Chang et al which found lower CCF in the CCC group.^25^ All the study protocols scored CCFs higher than 80% which is the cutoff value for optimal outcome recommended by the American Heart Association.

**Knowledge gaps** – All the studies retrieved in this scoping review are based on simulation trials on manikins. Consequently, the absence of literature based on human patients or animal models is the main knowledge gap. Moreover, given the importance that is attributed to rescue breaths in pediatric CPR, all models consider the event of OHCA in adult patients, therefore reducing the study population. Patients experiencing OHCA receiving bystander rescue are often given CPR by younger people.^6^ Even though it is discussed whether the rescuer’s physical training status is involved in the CPR performance,^40^ it should be taken into account that in most of the included studies participants were aged between 20 and 30 years, with the exception of the study from Rasmussen et al where participants median age was between 43 and 46 years.^26^ Considering a younger population of rescuers may bias the transferability of the obtained results to the general population. Therefore, studies including a wider cohort of participants with different age could enrich the existing evidence.

**Implications for research** – Future research should focus on improving the quality of the CPR delivered as well as incentivizing CPR initiation among laypeople in OHCA. CPR maneuvers ought to be evidence-based and easy to be memorized, performed and divulgated to laic people. Thus, when new protocols such as the inclusion of intentional interruptions are proposed, it is compulsory to thoroughly evaluate the pros and cons of simulation studies, animal models and clinical studies and, only afterward, consider the large-scale implementation of the changes. To date, the introduction of intentional interruptions during CO-CPR is still lacking evidence beyond simulation studies.

## Conclusions

The importance of minimally interrupted CPR in improving patients’ outcomes is well demonstrated by the existing literature. The inclusion of intentional interruptions during CO-CPR within the specific scenario of single rescuer bystander CPR has been investigated by simulation studies on manikins. There was variability among the proposed protocols. Findings are encouraging, with improved rate of compressions with correct depth and lower rating of rescuer’s perceived exertion while maintaining compression rate and chest compression fraction within guidelines. The quality of the available evidence is low and knowledge gaps should be filled by further adequately designed preclinical and clinical studies in order to draw more definitive conclusions on the applicability of these techniques. In this sense, further high-quality research and feasibility and safety of protocols incorporating intentional interruptions during CO-CPR seem to be justified.

## Authors' contributions

GC and AC conceived the content, drafted the manuscript, approved the final version to be submitted. MI, EB, MM, BS, SC, FRC and AG helped in writing the manuscript and revised it critically for important intellectual content, approved the final version to be submitted. All authors have agreed on the journal to which the article will be submitted, gave final approval of the version to be published, and agree to be accountable for all aspects of the work.

## CRediT authorship contribution statement

**Giulia Catalisano:** Writing – review & editing, Writing – original draft, Resources, Project administration, Methodology, Investigation, Formal analysis, Data curation, Conceptualization. **Marta Milazzo:** Writing – review & editing, Validation, Formal analysis, Data curation. **Barbara Simone:** Writing – review & editing, Validation, Formal analysis, Data curation. **Salvatore Campanella:** Writing – review & editing, Validation, Data curation. **Francesca Romana Catalanotto:** Writing – review & editing, Validation, Data curation. **Mariachiara Ippolito:** Writing – review & editing, Supervision, Methodology, Conceptualization. **Antonino Giarratano:** Writing – review & editing, Visualization, Validation. **Enrico Baldi:** Writing – review & editing, Visualization, Validation, Investigation, Data curation. **Andrea Cortegiani:** Writing – review & editing, Project administration, Methodology, Formal analysis, Conceptualization.

## Declaration of competing interest

The authors declare that they have no known competing financial interests or personal relationships that could have appeared to influence the work reported in this paper.
