# Supplementary material for: Intentional interruptions during compression only CPR: A scoping review
Source: Resusc Plus. 2024 Apr 4;18:100623. doi: 10.1016/j.resplu.2024.100623 (PMC11000192; doi:10.1016/j.resplu.2024.100623)
Supplement: Supplementary Data 1 [file mmc1.docx]

Search strategy

**EMBASE**

(**intermittent** OR **interruption** OR **interrupted** OR **pause** OR **'rest'**/exp OR **rest**) AND (**cpr** OR **'ohca'**/exp OR **ohca** OR **'out of hospital cardiac arrest'**/exp OR **'out of hospital cardiac arrest'** OR (**out** AND **of** AND (**'hospital'**/exp OR **hospital**) AND **cardiac** AND (**'arrest'**/exp OR **arrest**)) OR **'chest compressions'** OR ((**'chest'**/exp OR **chest**) AND **compressions**))

**Cinhal**

( Intermittent OR interrupted OR interruption OR Pause OR rest ) AND ( CPR OR OHCA OR out of hospital cardiac arrest OR chest compressions)

**Pubmed**

((Intermittent) OR (interrupted) OR (interruption) OR (pause) OR (rest)) AND ((CPR) OR (OHCA) OR (Out of hospital cardiac arrest) OR (chest compressions)) Sort by: First Author

("intermittant"[All Fields] OR "intermittence"[All Fields] OR "intermittencies"[All Fields] OR "intermittency"[All Fields] OR "intermittent"[All Fields] OR "intermittently"[All Fields] OR ("interrupt"[All Fields] OR "interrupted"[All Fields] OR "interrupting"[All Fields] OR "interruption"[All Fields] OR "interruptions"[All Fields] OR "interruptive"[All Fields] OR "interrupts"[All Fields]) OR ("interrupt"[All Fields] OR "interrupted"[All Fields] OR "interrupting"[All Fields] OR "interruption"[All Fields] OR "interruptions"[All Fields] OR "interruptive"[All Fields] OR "interrupts"[All Fields]) OR ("pause"[All Fields] OR "paused"[All Fields] OR "pauses"[All Fields] OR "pausing"[All Fields] OR "pausings"[All Fields]) OR ("rest"[MeSH Terms] OR "rest"[All Fields])) AND ("cardiopulmonary resuscitation"[MeSH Terms] OR ("cardiopulmonary"[All Fields] AND "resuscitation"[All Fields]) OR "cardiopulmonary resuscitation"[All Fields] OR "cpr"[All Fields] OR "OHCA"[All Fields] OR ("out of hospital cardiac arrest"[MeSH Terms] OR ("out of hospital"[All Fields] AND "cardiac"[All Fields] AND "arrest"[All Fields]) OR "out of hospital cardiac arrest"[All Fields] OR ("out"[All Fields] AND "hospital"[All Fields] AND "cardiac"[All Fields] AND "arrest"[All Fields]) OR "out of hospital cardiac arrest"[All Fields]) OR (("chested"[All Fields] OR "thorax"[MeSH Terms] OR "thorax"[All Fields] OR "chest"[All Fields] OR "chests"[All Fields]) AND ("compress"[All Fields] OR "compressed"[All Fields] OR "compresses"[All Fields] OR "compressibilities"[All Fields] OR "compressibility"[All Fields] OR "compressible"[All Fields] OR "compressing"[All Fields] OR "compression"[All Fields] OR "compression s"[All Fields] OR "compressions"[All Fields] OR "compressive"[All Fields] OR "compressively"[All Fields])))

**Scopus**

## (( intermittent  OR  interruption  OR  interrupted  OR  pause  OR  rest )  AND  ( cpr  OR  ohca  OR  out  AND  of  AND  hospital  AND  cardiac  AND  arrest  OR  chest  AND  compressions ) )
